# Active Coping Moderates the Link Between Neighborhood Segregation & Cognitive Decline in Black American Men

**DOI:** 10.1093/geroni/igaf122.1301

**Published:** 2025-12-31

**Authors:** Darlingtina Esiaka

**Affiliations:** University of Kentucky - College of Medicine, Lexington, Kentucky, United States

## Abstract

Black American men experience disproportionate exposure to neighborhood segregation, which may contribute to cognitive health disparities. Subjective cognitive decline (SCD) is an early indicator of potential cognitive impairment, yet protective factors such as active coping may buffer adverse effects. This study examines whether active coping moderates the association between neighborhood segregation and SCD in Black American men. Data were collected from a sample of 94 Black American men (Mean age = 43.71, SD = 8.59). A moderated regression analysis was conducted using the PROCESS Model 1 (Hayes, 2022) in SPSS V.30. The model examined the interaction between neighborhood segregation and active coping in predicting SCD while controlling for covariates. Neighborhood segregation was marginally associated with increased SCD (B = -0.94, p = .052) and active coping was significantly associated with lower SCD (B = -0.95, p = .008). A significant interaction between neighborhood segregation and active coping was observed (B = 0.03, p = .017), suggesting that the association between neighborhood segregation and SCD depends on active coping levels. At lower levels of active coping, neighborhood segregation was unrelated to SCD (p = .688). However, at moderate (p = .038) and high (p = .004) levels of active coping, greater neighborhood segregation was associated with increased SCD. Our findings highlight the complex interplay between neighborhood stressors and coping mechanisms in cognitive health. Also, the results suggest that while active coping may be protective in certain contexts, it may also exacerbate the cognitive health of people in segregated environments.

